# Microbial Regulation of Glucose Metabolism and Cell-Cycle Progression in Mammalian Colonocytes

**DOI:** 10.1371/journal.pone.0046589

**Published:** 2012-09-28

**Authors:** Dallas R. Donohoe, Aminah Wali, Bruna P. Brylawski, Scott J. Bultman

**Affiliations:** 1 Department of Genetics, University of North Carolina, Chapel Hill, North Carolina, United States of America; 2 Lineberger Cancer Center, University of North Carolina, Chapel Hill, North Carolina, United States of America; 3 Department of Pathology and Laboratory Medicine, University of North Carolina, Chapel Hill, North Carolina, United States of America; Charité-University Medicine Berlin, Germany

## Abstract

A prodigious number of microbes inhabit the human body, especially in the lumen of the gastrointestinal (GI) tract, yet our knowledge of how they regulate metabolic pathways within our cells is rather limited. To investigate the role of microbiota in host energy metabolism, we analyzed ATP levels and AMPK phosphorylation in tissues isolated from germfree and conventionally-raised C57BL/6 mice. These experiments demonstrated that microbiota are required for energy homeostasis in the proximal colon to a greater extent than other segments of the GI tract that also harbor high densities of bacteria. This tissue-specific effect is consistent with colonocytes utilizing bacterially-produced butyrate as their primary energy source, whereas most other cell types utilize glucose. However, it was surprising that glucose did not compensate for butyrate deficiency. We measured a 3.5-fold increase in glucose uptake in germfree colonocytes. However, ^13^C-glucose metabolic-flux experiments and biochemical assays demonstrated that they shifted their glucose metabolism away from mitochondrial oxidation/CO_2_ production and toward increased glycolysis/lactate production, which does not yield enough ATPs to compensate. The mechanism responsible for this metabolic shift is diminished pyruvate dehydrogenase (PDH) levels and activity. Consistent with perturbed PDH function, the addition of butyrate, but not glucose, to germfree colonocytes *ex vivo* stimulated oxidative metabolism. As a result of this energetic defect, germfree colonocytes exhibited a partial block in the G_1_-to-S-phase transition that was rescued by a butyrate-fortified diet. These data reveal a mechanism by which microbiota regulate glucose utilization to influence energy homeostasis and cell-cycle progression of mammalian host cells.

## Introduction

Metagenomics projects involving high-throughput/next-generation sequencing are being used to characterize microbial communities within the human body [1]–[9], yet our knowledge of how microbes influence biochemical and physiological processes in the host is limited. The gastrointestinal (GI) tract harbors the largest density of microbiota, where gut microbes outnumber all of the somatic cells and germ cells in the human body by 10-fold [10], [11]. Furthermore, if we consider the their collective genomes, referred to as the microbiome, the microbiota have approximately 100 times more genes than our own human genome [12]. Current sequence data shows the gut microbiome is enriched for genes involved in energy production and extraction [3], [6], [7]. For example, carbohydrate metabolism is particularly overrepresented with 115 families of glycoside hydrolases and 21 families of polysaccharide lyases [3], [13]. Humans lack many of these genes presumably because mammals (and their genomes) co-evolved with gut microbiota (and the gut microbiome) [12], [14]–[17]. As a result, there is a symbiotic relationship where humans and other mammalian hosts ingest carbohydrate-rich diets and provide them to microbiota in a protected environment; in return, the microbiota break down the glycans into disaccharides and monosaccharides for their own use as well as the host [18], [19]. Because glycans are poorly digested by the host, microbiota are thought to improve the ability of the host to absorb nutrients and extract calories from their diet [18], [20].

To study the function of microbiota in host metabolism, germfree (GF) mice, which completely lack microbes, can be compared to genetically identical mice that were raised conventionally (CONV-R) with “normal”, albeit undefined, microbiota. Previous studies have shown that GF mice consume more food to maintain the same body weight as CONV-R controls [21], [22]. Despite this increased food intake, GF mice are leaner than CONV-R controls and are resistant to obesity induced by a high-fat diet [21]. They also have decreased levels of glycogen, glucose, and insulin [21]. These findings support the idea that microbiota increase metabolic efficiency in the host.

In a previous study, we reported that colonic tissue derived from GF mice exhibited diminished NADH/NAD^+^ ratios and ATP levels as compared to colonic tissue derived from CONV-R mice [23]. Other tissues surveyed, however, showed no difference in these same energetic biomarkers between GF and CONV-R mice, thereby suggesting a selective role of microbiota in regulating energy homeostasis in the colon. The most likely reason for this tissue specificity is that colonocytes utilize bacterially-produced butyrate as their primary energy source [24], [25], whereas most other cell types rely on glucose. Butyrate is a short-chain fatty acid (SCFA) produced by bacterial fermentation of fiber in the lumen of the proximal colon, and we demonstrated that butyrate rescues a severe deficit in oxidative metabolism in GF colonocytes [23]. However, we were surprised that glucose did not compensate for the lack of butyrate in GF colonocytes because most or all cells have the capacity to metabolize glucose. Here, in this study, we address this issue experimentally by analyzing glucose uptake and metabolism in GF and CONV-R colonocytes. We also demonstrate that the perturbed metabolic state of GF colonocyte results in a partial block in the cell-cycle as compared to CONV-R colonocytes, and that feeding GF mice a butyrate-fortified diet incompletely rescue this cell-cycle block in the GF colonocyte.

## Results

### Effect of Microbiota on Energy Homeostasis in Different Segments of the GI Tract

**Figure 1:**
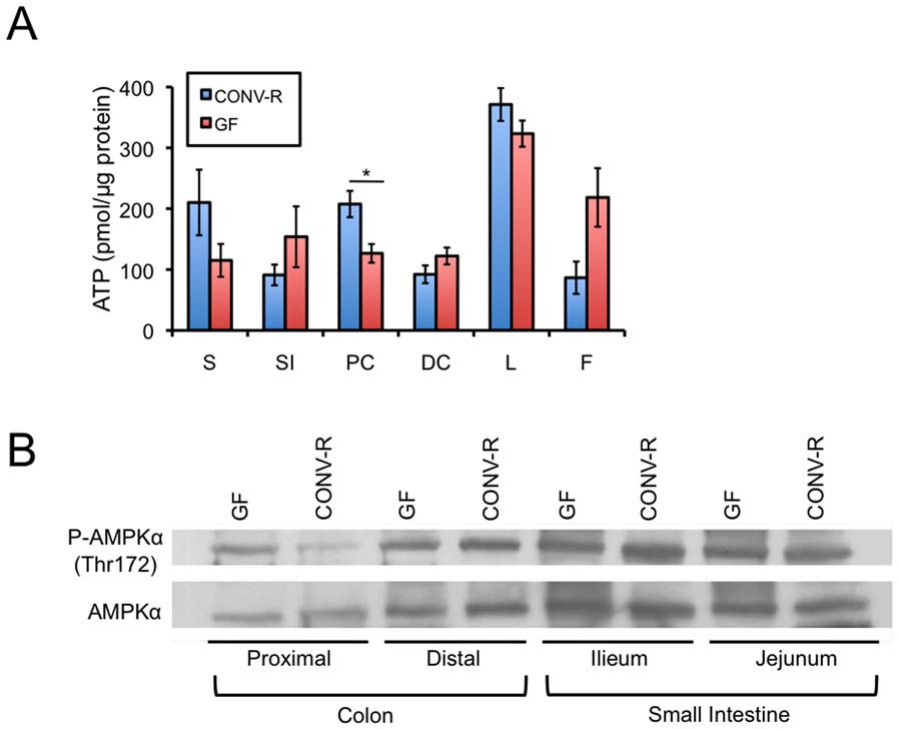
Effects of microbiota on energy metabolism biomarkers in the GI tract and digestive tissues. A, ATP levels in the stomach (S), small intestine (SI), proximal colon (PC), distal colon (DC), liver (L), and fat (F) (white adipose tissue) of CONV-R and GF mice. Five CONV-R and five GF mice were analyzed, and the results are mean ± standard error. Significant differences are indicated (*p<0.05). B, Western blot analysis of AMPKα phosphorylated at Thr172 (top panel) and total AMPKα (bottom panel) in different segments of the colon and small intestine from GF and CONV-R mice. Results are representative of 3 independent experiments.

We previously demonstrated that microbiota are required for normal ATP levels in the colon but not in several other tissues that were tested [23]. Here, we extended this analysis to different segments of the GI tract because it is known to harbor the highest densities of microbes in the body. In addition to the stomach, small intestine (jejunum and ileum), proximal colon, and distal colon, we also analyzed liver and fat (white adipose tissue) because of their important roles in digestion and metabolism. The proximal colon, but none of the other tissues that were tested including the distal colon, had significantly diminished ATP levels in GF mice compared to CONV-R controls (Figure 1A). AMPKα (5′-adenosine monophosphate-activated protein kinase a) is a metabolic sensor activated by low ATP levels relative to AMP. Therefore, we evaluated AMPKα activation status based on increased Thr172 phosphorylation. As expected, we observed an increase in AMPKα activation in the proximal colon of GF mice relative to CONV-R but not in the distal colon or small intestine (Figure 1B). These results extend our previous findings [23] showing that the energetic deficit is not observed in all GI segments, and support the idea that the proximal colon is particularly dependent on microbiota.

### GF Colonocytes Exhibit Increased Glucose Uptake and Glycolysis

**Figure 2:**
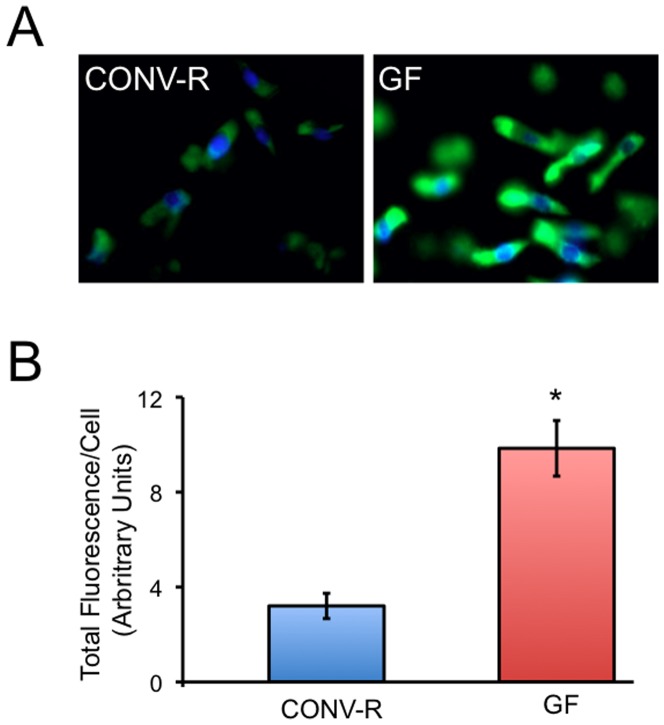
Microbial regulation of glucose uptake. A, 2-deoxyglucose uptake observed as green fluorescence in CONV-R (left) and GF (right) colonocytes counterstained with DAPI (blue). B, Quantification of total 2-deoxyglucose fluorescence per cell in CONV-R and GF colonocytes. Results are based on 20 cells per colonocyte preparation from 3 independent mice and represent the mean ± SE with significant differences indicated (*p<0.01).

To understand the effects of microbiota on colonocyte metabolism, we analyzed our previous transcriptome profile data comparing freshly isolated colonocytes from CONV-R and GF mice [23]. This analysis revealed that cell-surface transporters were among the most highly enriched gene products upregulated in GF colonocytes. For example, 26 members of solute carrier (SLC) families were upregulated, whereas only 7 were downregulated (Table S1). The SLC2 family encodes facilitative (ATP-independent) glucose transporters (GLUTs), and 3 out of 13 members were upregulated in GF (Table S1). Most GLUTs are not expressed ubiquitously so having a subset of them affected was expected. The SLC5A family encodes active (ATP-dependent) glucose transporters. Members of this family are also expressed in a tissue-restricted manner, and 2 out of 12 were upregulated in GF including one (SLC5A1) known to function in the intestine (Table S1). Members of other SLC families that were upregulated are known to take up other energy substrates such as fatty acids and amino acids (Table S1). These findings suggest that GF colonocytes attempt to compensate for the lack of butyrate by increasing their uptake of glucose and other alternative energy substrates. To investigate whether the increased expression of glucose transporters is functionally relevant and results in increased glucose uptake, we isolated colonocytes from CONV-R and GF mice in the presence of 6-[N-(7-nitrobenz-2-oxa-1,3-diazol-4-yl) amino]-2-deoxy-d-glucose (6-NBDG), which is a fluorescent glucose analog, and analyzed its uptake *ex vivo*. Confocal microscopy was performed within an hour of colonocyte isolation, and 6-NBDG levels were 3.5-fold higher inside of GF colonocytes than CONV-R controls (Figure 2).

**Figure 3:**
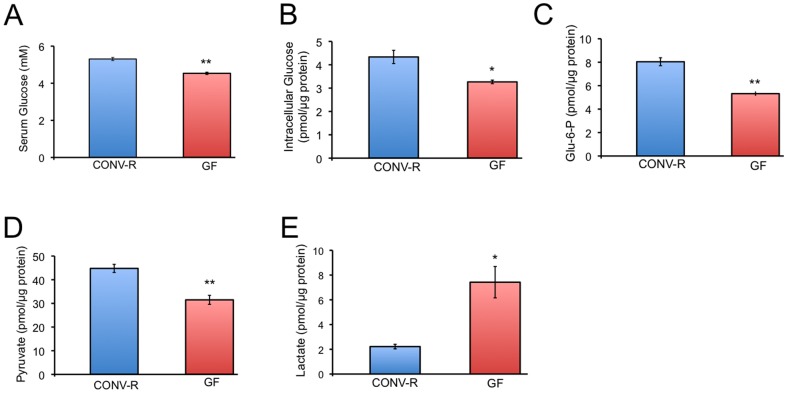
Microbial regulation of glycolysis in colonocytes. A–E, Levels of serum glucose (A), intracellular glucose (B), glucose-6-phosphate (Glu-6-P) (C), pyruvate (D), and lactate (E) in CONV-R and GF serum (A) or colonocytes (B–E). Results are mean ± standard error from 3 independent experiments with significant differences indicated (*p<0.05; **p<0.01).

6-NBDG cannot be oxidized, which makes it ideal for measuring glucose uptake but not suitable for measuring glycolysis. To evaluate glycolysis, it was first necessary to analyze substrate availability so we measured glucose levels in the serum of CONV-R and GF mice. Similar to a previous finding [21], we observed a mild hypoglycemia in GF mice compared to CONV-R controls (Figure 3A). Next, we measured intracellular glucose, two key intermediates of glycolysis (glucose-6-phosphate and pyruvate), and lactate (the final end product of glycolysis) inside of colonocytes. GF colonocytes had a ∼25% decrease in intracellular glucose, glucose-6-phosphate, and pyruvate levels compared to CONV-R, whereas lactate levels were increased 3.5 fold compared to CONV-R (Figure 3B–E). These results suggest that glucose is taken up by GF colonocytes in greater quantities but it does not accumulate because the rate of glycolysis is also increased to an extent that yields a commensurate increase in lactate.

### Glucose is Not Metabolized Oxidatively by GF Colonocytes

**Figure 4:**
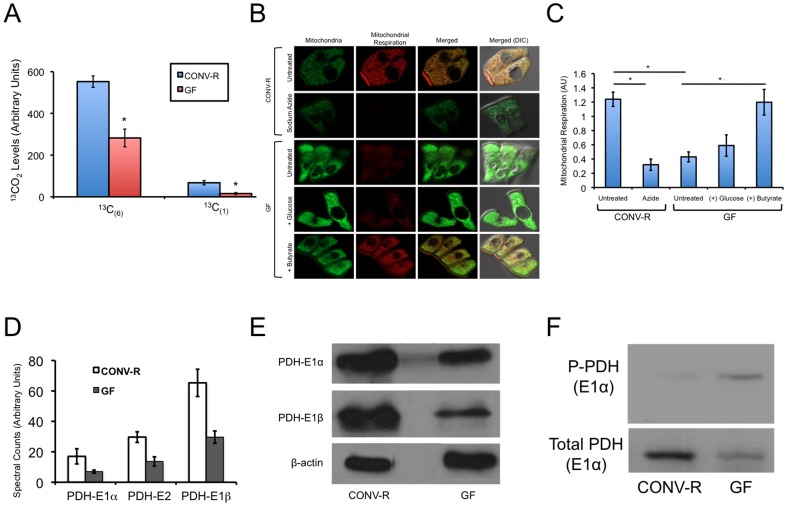
Microbes and butyrate stimulate oxidative metabolism. A, Levels of free ^13^CO2 released from CONV-R and GF colonocytes following their incubation with uniformly labeled (^13^C_(6)_) or singly labeled (^13^C_(1)_) glucose. ^13^CO2 levels were detected by isotope-ratio mass spectrometry, and the results are presented as mean ± standard error from 3 independent experiments with significant differences indicated (*p<0.01). B, Oxidative metabolism indicated by MitoTracker Red CM-H_2_XRos (red fluorescence; second column) and total mitochondria indicated by the non-oxidizable MitoTracker Green FM (green fluorescence; first column) in colonocytes from CONV-R (top two rows) and GF (bottom three rows). The sodium azide treated cells in the second row serve as a negative control. The effect of glucose and butyrate on GF colonocytes in the two bottom rows is compared to untreated GF colonocytes in the row immediately above. C, Quantification of oxidative metabolism in the different experimental groups. Determined as the ratio of red fluorescence relative to green fluorescence (internal control). Results are based on 20 cells per condition from 3 independent experiments and represent the mean ± SE with significant differences indicated (*p<0.01). D, Spectral counts from quantitative mass spectrometry of pyruvate dehydrogenase subunits in CONV-R and GF colonocytes. E, Western blot analysis of the E1α and E1b subunits of PDF from CONV-R and GF colons with β–actin serving as a loading control. Results are representative of 3 independent experiments. F, Western blot analysis of phospho-PDH (E1α) and total PDH (E1α) from CONV-R and GF colons. Results are representative of 3 independent experiments.

When a single molecule of glucose is metabolized by glycolysis to lactate, it yields only 2–4 ATPs; in contrast, when glucose is converted to pyruvate followed by oxidative metabolism inside mitochondria, it yields 36 ATPs. Considering that GF colonocytes are in a suboptimal energetic state, it is surprising that they metabolize glucose to lactate rather than oxidatively to CO_2_. To demonstrate that oxidative metabolism is, in fact, diminished in GF colonocytes, we performed flux experiments by adding uniformly labeled ^13^C-labeled glucose to primary colonocytes as they were being isolated from CONV-R and GF mice and then measuring ^13^C-labeled CO_2_ using gas isotope ratio mass spectrometry. These experiments demonstrated that in GF colonocytes, ^13^C_(6)_-glucose is oxidized to ^13^CO_2_ at only 51% of the normal oxidation rate that occurs in CONV-R colonocytes (Figure 4A).

Diminished CO_2_ production should be coupled to diminished oxidative phosphorylation, which we analyzed by incubating primary colonocytes with two mitotracker probes simultaneously. One probe fluoresced red upon being oxidized and provided a measure of oxidative phosphorylation, while the other probe fluoresced green regardless of mitochondrial oxidative activity and therefore served as an internal control. As described previously [23], GF colonocytes exhibited a marked decrease in mitochondrial oxidative activity (red in row 3, column 2) but showed no difference in total mitochondria (green in row 3, column 1) (Figure 4B). The extent to which oxidative phosphorylation was diminished in GF colonocytes is striking because it was more similar to sodium azide treated negative controls (red in row 2, column 2) than CONV-R samples (red in top row, column 2). Next, we added glucose to GF colonocytes and observed only a modest increase in oxidative metabolic activity (red in row 4, column 2) (Figure 4B,C). In contrast, when we added butyrate to GF colonocytes, it stimulated such a large increase in oxidative metabolic activity that it was comparable to CONV-R controls (red in bottom row, column 2) (Figure 4B,C). These results indicate that although GF colonocytes are competent to undergo oxidative metabolism (based on the short-chain fatty acid butyrate), they metabolize glucose primarily by glycolysis rather than oxidatively via the TCA cycle and oxidative phosphorylation.

### PDH Inhibition Prevents Glucose from being Oxidized by GF Colonocytes

Because butyrate stimulates oxidative activity in GF colonocytes, the mitochondrial enzymes involved in the TCA cycle and electron transport chain must be functional. Therefore, the inability of glucose to stimulate robust oxidative metabolism must be due to an alteration specific to glucose metabolism (i.e., not involving enzymes that also participate in butyrate metabolism). We previously performed a proteomic analysis to identify protein expression differences between CONV-R and GF colonoytes [23]. Further analysis of these data revealed a significant decrease in the levels of the E1α, E1β, and E2 subunits of the pyruvate dehydrogenase (PDH) complex in GF compared to CONV-R colonocytes (Fig. 4*D*). PDH is a particularly good candidate because it facilitates oxidative glucose metabolism by converting cytosolic pyruvate to mitochondrial acetyl-CoA immediately upstream of the TCA cycle [26], but is not involved in fatty acid metabolism. Accordingly, downregulation of PDH would be expected to result in decreased oxidative metabolism of glucose and a concomitant rise in glycolysis (i.e., lactate production). To validate the mass spectrometry results, we performed western blot analyses for PDH-E1a and PDH-E1b subunits and found that their level of expression was markedly diminished in GF colonocytes compared to CONV-R colonocytes (Figure 4E). We hypothesized that PDH activity would also be diminished in GF because of increased mRNA levels of pyruvate dehydrogenase kinase 2 (*Pdk2*) (based on analysis of our previous transcriptome data [23]), which phosphorylates and inactivates the E1α regulatory subunit [26]. To test this hypothesis, we analyzed levels of PDH-E1a phosphorylated at Ser293. Despite total PDH-E1a being diminished, phospho-PDH-E1a was increased (Figure 4E). Low levels of PDH-E1a combined with the fact that a relatively large percentage of the molecules are inactivated by phosphorylation suggested that PDH activity should be diminished in GF colonocytes. To test this hypothesis, we performed flux experiments using singly-labeled glucose (^13^C_(1)_-glucose) rather than uniformly labeled glucose (^13^C_(6)_-glucose) because the first CO_2_ that is released (in this case as ^13^CO_2_) is due to PDH. Analysis of CONV-R and GF colonocytes incubated with ^13^C_(1)_-glucose revealed that PDH activity was diminished in GF by 94% (Figure 4A). Taken together, these experiments provide an explanation for why GF colonocytes undergo increased glycolysis and decreased oxidative metabolism.

### GF Colonocytes Have a Cell-Cycle Defect

The perturbed energy metabolism of GF colonocytes might be expected to have deleterious consequences, and we previously determined that these cells undergo increased autophagy [23]. Based on our analysis of transcriptome profiles, we also identified decreased expression of many histone genes. The four canonical histones (H2A, H2B, H3, and H4) are each represented by 12–17 genes arranged in 3 clusters [27]. The largest of these clusters (*Hist1*) covers a large genomic interval (∼2 Mbp) and contains the majority of the core histone genes plus all 6 of the linker histone (H1) genes. Out of 64 histone genes, 27 were downregulated in GF while none were upregulated (Table S2). The extent of downregulation averaged 1.7 fold with a range of 1.4 to 2.1 (Table S2). Because each of these histone genes is transcribed only during DNA replication [28], this finding suggested that a lower percentage of GF colonocytes are in S phase than CONV-R colonocytes.

**Figure 5:**
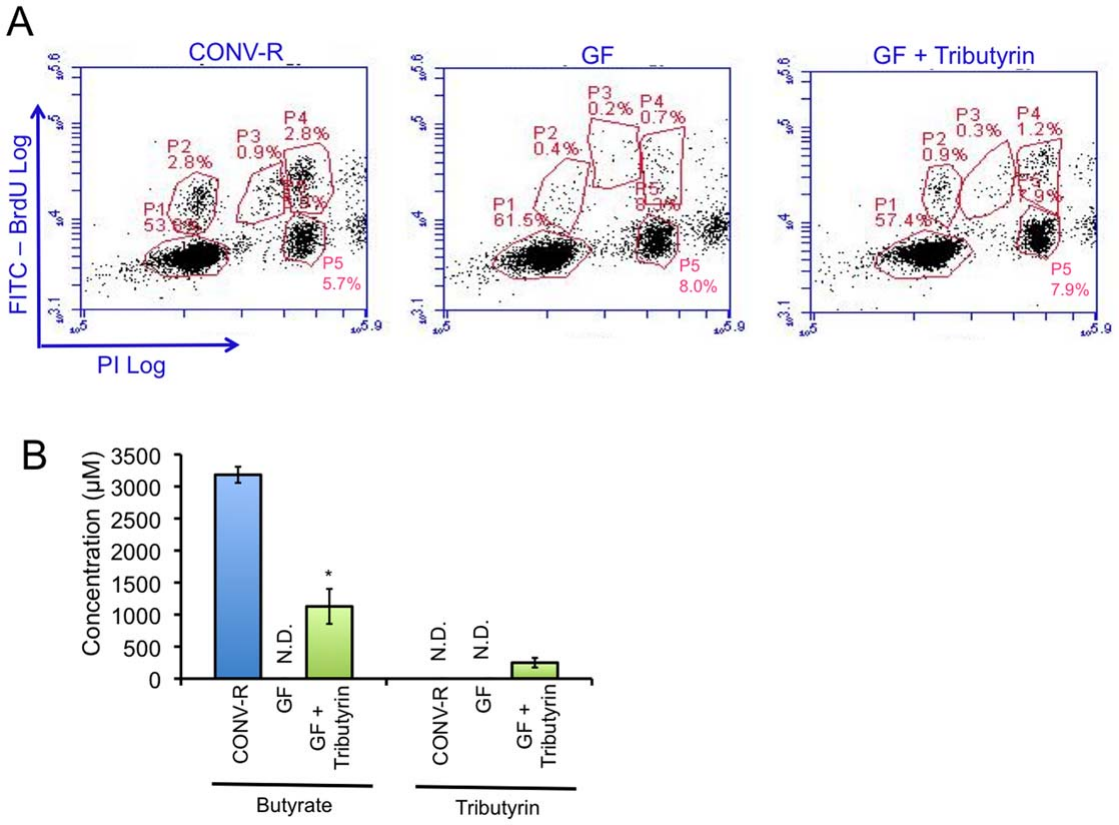
Microbes and butyrate promote cell-cycle progression at the G1-to-S phase transition. A, Flow cytometry profiles of colonocytes from CONV-R mice (left), GF mice (center), and GF mice provided a tributyrin-fortified diet (right). PI levels are shown on the x-axis, and BrdU incorporation is shown on the y-axis. The percentage of cells in various stages of the cell cycle are indicated: P1, G_1_; P2, early S; P3, middle S; P4, late S; P5, G_2_. Results are representative of 3 independent experiments. B, Measurements of luminal butyrate (left) and tributyrin (right) levels from the colon of CONV-R mice and GF mice provided either a provided a control diet (GF) or a tributyrin-fortified diet (GF + Tributyrin). Results are mean ± standard error from 3 independent experiments with significant differences indicated (*p<0.01). N.D., not detectable.

To confirm that the histone genes were serving as an accurate marker of S phase and that GF colonocytes have a cell-cycle defect, we performed flow cytometery to quantify the percentage of cells in various stages of the cell cycle. Using BrdU to detect cells in S phase and propidium iodide (PI) as a measure of DNA content, these experiments demonstrated that GF mice have an ∼80% reduction in colonocytes undergoing DNA replication in S-phase (Figure 5A). 1.3% of GF colonocytes were in S phase compared to 6.5% for CONV-R (Figure 5A). There was no significant difference in the distribution of cells in early versus late S phase suggesting DNA replication progression is not perturbed. However, 61.5% of GF colonocytes were in G_1_ compared to 53.8% for CONV-R controls. The increased percentage of GF colonocytes in G_1_ coupled with the decreased percentage in S phase suggests there was a partial block in the transition from G_1_ to S phase. This result was consistent in three independent experiments and suggests that the suboptimal energetic state of GF colonocytes decreased cell-cycle progression at the G_1_ to S phase transition. The GF samples also showed an increased percentage of cells in G_2_ suggesting that there was also a partial block at the G2 to M phase transition, which represents the other major cell-cycle checkpoint.

Although the above findings demonstrate the importance of microbiota in cell-cycle progression, they do not address whether butyrate is a causal factor. Therefore, we provided GF mice with an identical diet that was calorically matched except it was fortified with tributyrin. Tributyrin is more stable than butyrate (i.e., less is absorbed in the small intestine before it reaches the colon) and is converted into 3 butyrate molecules in the colon. Our LC-MS/MS analysis of luminal contents of these mice confirmed that tributyrin was efficiently converted to butyrate in the colon based on the concentration of tributyrin being only ∼25% of butyrate (Figure 5B). Whereas butyrate was not detectable in the colon of GF mice provided the control diet, butyrate levels were greater than 1 mM in GF mice provided the tributyrin-containing diet. This level was 36% of that measured in CONV-R mice (Figure 5B). Cell-cycle analysis of GF colonocytes using BrdU and PI showed that the tributyrin diet partially rescued the G_1_-to-S phase block (Figure 5A). The percentage of cells in S phase (2.4%) and in G_1_ (57.4%) was intermediate between the CONV-R and GF samples. This rescue is consistent with our finding that butyrate stimulates oxidative metabolism in GF colonocytes (Figure 4B,C) and identifies butyrate as bacterial metabolite that can promote cell-cycle progression of mammalian host cells.

## Discussion

**Figure 6:**
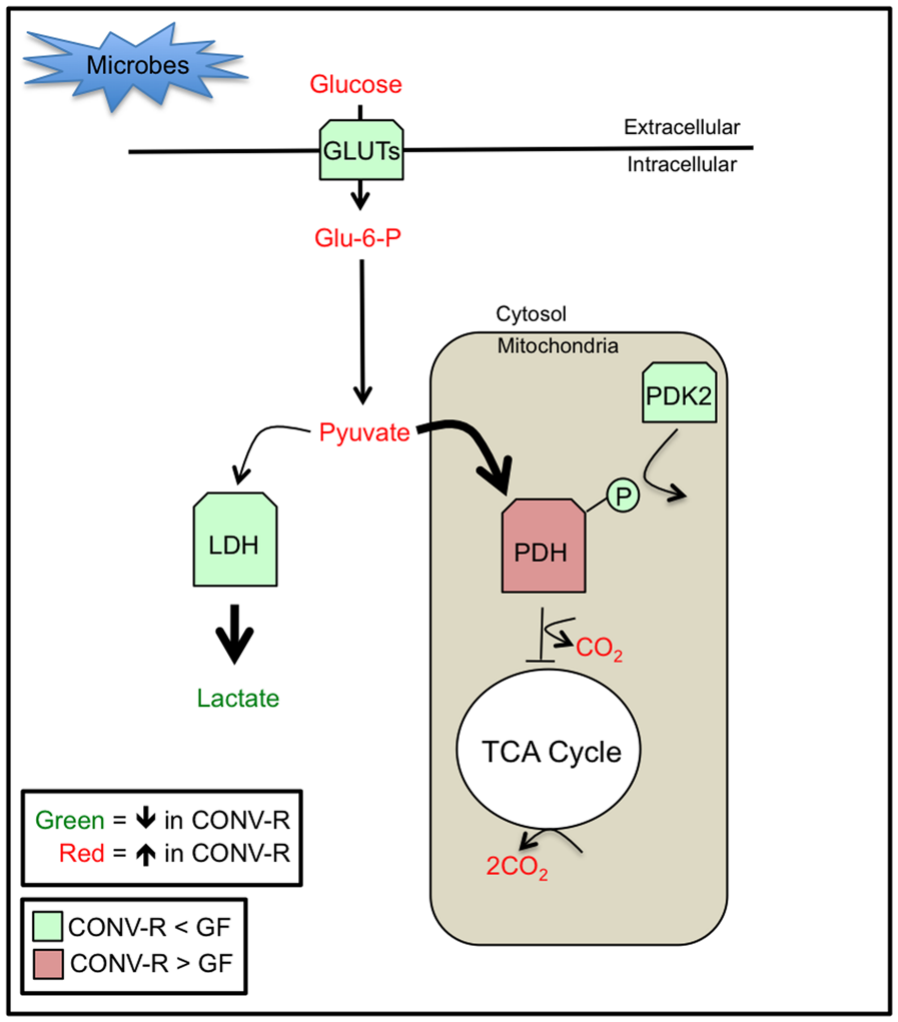
A model of microbial regulation of glucose metabolism in host colonocytes. Microbes increase blood glucose levels and increase PDH levels and activity to facilitate oxidative metabolism of glucose in the mitochondria. In contrast, GF mice are hypoglycemic, and their colonocytes respond by increased expression of GLUTs. Although this leads to increased glucose uptake, Glu-6-P and pyruvate levels are diminished because of their conversion to lactate, which is increased in GF colonocytes. This increase in glycolysis can be attributed to decreased PDH expression/activity and decreased oxidative metabolism in the mitochondria. Not only are PDH levels decreased, but also enzymatic activity is inhibited via phosphorylation of the E1a regulatory subunit due to increased expression of PDK2 in GF colonocytes.

In this study we demonstrate that microbiota promote glucose metabolism at two levels. At a systemic level, microbiota increase blood glucose levels since GF mice are mildly hypoglycemic, which is consistent with a previous report [21]. This is consistent with the idea that the gut microbiome is highly enriched for genes encoding glycoside hydrolases and polysaccharide lyases that convert glycans into simple sugars that are absorbed in the small intestine. At a local level, microbiota also influence how glucose is utilized by colonocytes, and this is presented as a model in Figure 6. Based on comparisons between CONV-R and GF colonocytes, microbes promote oxidative metabolism by increasing PDH levels and enzymatic activity to direct glucose-derived carbon into the TCA cycle. This is apparent from the results of our experiments showing that GF colonocytes take up increased levels of glucose but shift away from oxidative metabolism/CO_2_ production and towards glycolysis and lactate production. Although this metabolic shift apparently does not occur in GF liver [28], it might occur in other GF tissues since GF mice consume less oxygen than CONV-R controls [21]. The increased glucose uptake in GF colonocytes presumably represents an attempt to compensate for the loss of their preferred energy substrate, which is bacterial-produced butyrate. However, the downregulation of PDH and shift to glycolysis does not yield enough ATPs to compensate. Unlike tumor cells undergoing the Warburg effect, it is not clear how this shift to glycolysis would be advantageous to GF colonocytes. Considering that ATP levels of GF colonocytes are approximately half of CONV-R controls, this shift from oxidative metabolism to glycolysis might be viewed as a pathological condition that prevents increased glucose uptake from compensating. The mammalian genome co-evolved with the gut microbiome so it is not surprising that metabolic defects arise in the GF condition.

The decreased ATP levels in GF colonocytes leads to AMPK activation as shown here and autophagy as shown previously [23]. One might expect decreased ATP levels to result in additional deleterious consequences, and here we demonstrate that cell-cycle progression is diminished at the transition from G_1_ to S phase. One possibility is that a nutrient sensing pathway, possibly involving AMPK, regulates the restriction point of the cell cycle. However, other possibilities cannot be excluded. For example, we did not observe energy homeostasis perturbations in the GF small intestine, yet this tissue also shows diminished cell proliferation based on previous studies that measured [^3^H]-thymidine incorporation in GF samples compared to CONV controls [29], [30]. However, in our experiments, we know that butyrate is a causal factor because providing GF mice with tributyrin partially rescued the G1-to-S phase block. Because butyrate is the preferred energy source of colonocytes, our work supports the idea that the cell-cycle defect in GF colonocytes arises because of suboptimal energy metabolism.

## Materials and Methods

### Ethics Statement and mice

All mouse experiments were approved by the Institutional Animal Care and Use Comittees (IACUC) review board at the University of North Carolina as approved protocol ID # 10-186 and were performed in accordance with federal guidelines. Eight- to fourteen-week-old CONV-R or GF male C57BL/6 mice were used in all experiments, with the exception of ATP experiments, which used BALB/c mice. Conventionally-raised mice were reared in a specific pathogen-free (SPF) facility, while germfree mice were maintained in a gnotobiotic facility. Light/dark cycles and animal husbandry practices were identical between the facilities except that aseptic technique was performed in the gnotobiotic facility. For the flow cytometry experiments, the tributyrin-fortified (5AVC) and control (5SRZ) diets were from Test Diet (Richmond, IN).

### Isolation of Colonocytes

Colonic epithelial cells were isolated according to [31] in order to exclude cell types other than colonocytes such as immune cells, smooth muscle, and enteric neurons. Briefly, animals were euthanized by CO_2_ asphyxiation followed by cervical dislocation. Proximal colons were dissected, flushed with sterile Phosphate-buffered Saline (PBS), and splayed open longitudinally along Whatmann paper. The colon was then washed 3 more times in PBS, pealed from paper, and placed in PBS solution containing 5 mM EDTA. Submerged colon was then incubated on a rotator for 30 minutes at 37°C. After 30 minutes, colonic tissue was removed leaving isolated colonocytes, which were washed twice with PBS, and used for experiments.

### Western Blot

Cells or tissues were flash-frozen and homogenized or agitated in 8.3 M urea lysis buffer with NaF and Na orthovanadate and incubated on wet ice for 30 minutes. Samples were then centrifuged at 14,000 rpm for 20 minutes at 4°C. Protein supernatant was transferred to a new 1.5 mL eppendorf tube and protein concentration was determined by Bradford assay. Protein homogenates were separated on 10, 12, and 4–20% gradient SDS-polyacrylamide gels and transferred onto PVDF membrane. Membranes were then incubated on rocker in 3% BSA or 5% non-fat dry milk with NaF for 1 hr at RT. Blocked membranes were incubated in 1°Ab overnight on rotator at 4°C. Antibodies were used at 1∶250 to 1∶2000 and included AMPKα (Cell Signaling, Danvers, MA), phospho-AMPKα (Cell Signaling), pyruvate dehydrogenase cocktail (Mitosciences, Eugene, OR; MSP02), phospho-PDH-E1a (Abcam; ab92696), and β-actin (Abcam; ab8226). Blots were washed 3X for 10 min/wash in 1X TBST or 1X PBST (for non-phospho Westerns) at RT and incubated with horseradish peroxidase-conjugated secondary antibodies (1∶2000 to 1∶4000) for 1 hr and 30 min on rocker at room temperature. Membranes were then washed 3 additional times in 1XTBST or 1XPBST (for non-phospho Westerns), treated with ECL reagent, and developed for analysis.

### Metabolic Assays

All metabolic assays were performed with Biovision (Mountain View, CA) metabolism kits. Specifically, ATP (K354-100), glucose (K606-100), glucose-6-phosphate (K657-100), pyruvate (K609-100), and lactate (K607-100) were used in accordance to manufacturer specifications. For all assays, flash-frozen tissue was deproteinized with Perchloric acid kit (K808-200), which was also from Biovision. Results represent 4–6 independent biological replicates.

### Fluorescence Microscopy

The reagents 6-[N-(7-nitrobenz-2-oxa-1,3-diazol-4-yl) amino]-2-deoxy-d-glucose (6-NBDG), MitoTracker Red CM-H_2_XRos, and MitoTracker Green FM were purchased from Invitrogen (Carlsbad, CA). For 6-NDBG uptake assessment, 1 µM 6-NDBG was added to PBS/EDTA solution while isolating colonocytes (see above). Colonocytes were stained with DAPI and mounted on slides with coverslip. Cells were viewed with Olympus IX81 microcope equipped with FITC, Texas Red, Rhodamine, and DAPI filters. All fluorescent micrographs were acquired with the same exposure and focal plane. Image J (Bethesda, MD) was used to quantify integrated density (total fluorescence) per cell.

In a manner similar to the 6-NDBG, mitotracker dyes were added at 500 nM (MitoTracker Red CM-H_2_XRos), and 500 nM (MitoTracker Green FM) while isolating colonocytes. Image J was used to analyze mean fluorescence for both mitotracker dyes. The red-to-green fluorescence is an indication of oxidative metabolism in the colonocytes. All micrographs were taken with identical exposures. Z-stacks were acquired for each image and for each cell the individual z-stacks did not differ in red-to-green mean fluorescence between focal planes. For both 6-NDBG and mitotracker experiments, we used 3 independent biological replicates per experimental group.

### Liquid Chromatography - Mass Spectrometry

GF mice were given a tributyrin-fortified diet over a two-week period, Endogenous butyrate and tributyrin were measured from luminal contents of CONV-R and GF mice. An internal ^13^C_1_-butyrate standard was added, and luminal contents were resuspended in 0.1% ammonium hydroxide. Proteins were removed by centrifugation through 3-kDa spin-filters. Flow through was then analyzed for butyrate or tributyrin content by HPLC separation with subsequent detection by an Agilent 6520 AccurateMass Q-TOF mass spectrometer operating in negative mode (Santa Clara, CA). Peak areas were calculated using MassHunter Workstation software. Chromatographic peaks were integrated for samples and areas were compared to peak area for standards (10 µM) for each compound.

### Isotope Ratio Mass Spectrometry

The amount of ^13^CO_2_ in Exetainer breath storage tubes generated from colonocytes was measured with a Europa Scientific 20/20 gas isotope ratio mass spectrometer (Europa Scientific, Cincinnati, OH). Dissolved CO_2_ in solution was liberated to the tube headspace by addition of 100 mL saturated citric acid. The ratio of ^13^CO_2_ to ^12^CO_2_ (mass 45 to 44) was measured directly from the sample tube headspace. All samples were compared to an internal reference gas (5% CO_2_, balance 75% N_2_, 20% O_2_) that had been calibrated against the international standard PeeDeeBelmnite (PDB). Changes in ^13^CO_2_ enrichment before and after glucose administration were calculated. CO_2_ standards at three different ^13^C enrichments were run before and after the run to check instrument performance. The analytical precision of the instrument is 0.0001 atom % ^13^C.

### Flow Cytometry

Mice received i.p. injections of BrdU (50 mg/kg body weight) and colonocytes were prepared (as described above) 2 hours later. Following wash steps, 2×10^6^ colonocytes in 1 mL of PBS were fixed by the addition of 3 mL of ice-cold ethanol. This was done dropwise while vortexing. Following fixation, 1 mL of 50 mg/mL propidium iodide and 50 mL of 10 mg/mL of RNAse A was added and incubated at 4°C for 3 hours. For flow cytometry, mouse anti-BrdU followed by goat-anti-mouse IgG FITC incubation/wash steps were followed by flow cytometry following standard procedures.

### Statistics

For all metabolic assays and image quantification (mitotracker and 6-NDBG experiments) significant differences between experimental groups were determined by two-tail t-test (p<0.05). All data are expressed as mean ± SEM.

## Supporting Information

Table S1 SLC Gene Expression Changes in GF Colonocytes. Data are derived from a transcriptome analysis from CONV-R and GF colonocytes.
(PDF)

Table S2 Histone Gene Expression Changes in GF Colonocytes.
(PDF)
