# Supplementary material for: Microbial Regulation of Glucose Metabolism and Cell-Cycle Progression in Mammalian Colonocytes
Source: PLoS One. 2012 Sep 28;7(9):e46589. doi: 10.1371/journal.pone.0046589 (PMC3460890; doi:10.1371/journal.pone.0046589)
Supplement: Table S1 — SLC Gene Expression Changes in GF Colonocytes. Data are derived from a transcriptome analysis from CONV-R and GF colonocytes. (PDF) [file pone.0046589.s001.pdf]

TABLE 1
SLC gene expression changes in GF colonocytes

Gene	Solute carrier family	Status in GF	Fold change
Slc1a1	glutamate, neutral amino acids	downregulated	3.0
Slc1a4	glutamate, neutral amino acids	upregulated	1.7
Slc1a5	glutamate, neutral amino acids	upregulated	1.5
Slc2a6	glucose (facilitative GLUTs)	upregulated	2.3
Slc2a9	glucose (facilitative GLUTs)	upregulated	1.5
Slc3a1	heteromeric amino acids	upregulated	3.4
Slc5a1	sodium glucose (active GLUTs)	upregulated	1.5
Slc5a6	sodium glucose (active GLUTs)	upregulated	1.5
Slc6a6	Na^+-, Cl^--dependent neurotransmitter	upregulated	1.6
Slc6a19	Na^+-, Cl^--dependent neurotransmitter	upregulated	1.7
Slc7a9	cationic amino acid/glycoprotein	upregulated	3.4
Slc9a3	Na^+/H^+ exchanger	upregulated	1.7
Slc9a3r1	Na^+/H^+ exchanger	upregulated	1.5
Slc9a3r2	Na^+/H^+ exchanger	upregulated	1.4
Slc11a2	proton-coupled metal ion	upregulated	1.4
Slc12a8	electroneutral cation-Cl	upregulated	2.2
Slc15a2	proton oligopeptide	upregulated	1.4
Slc9a3	Na^+/H^+ exchanger	upregulated	1.7
Slc16a5	monocarboxylate (incl SCFAs)	upregulated	1.5
Slc17a5	vesicular glutamate	upregulated	1.8
Slc21a2	organic anion	upregulated	2.0
Slc23a3	Na^+-dependent ascorbic acid	upregulated	1.5
Slc24a6	$Na^+/(Ca^{2+}$-$K^+)$ exchanger	downregulated	1.5
Slc26a1	multifunctional anion exchanger	downregulated	2.0
Slc26a6	multifunctional anion exchanger	upregulated	1.9
Slc27a4	fatty acid	upregulated	1.4
Slc29a2	facilitative nucleoside	upregulated	1.4
Slc30a6	zinc efflux	downregulated	1.4
Slc37a1	sugar/phosphate exchanger	downregulated	1.6
Slc38a2	sodium coupled neutral amino acid	upregulated	1.5
Slc39a4	metal ion	downregulated	2.4
Slc39a10	metal ion	downregulated	1.6
Slc39a14	metal ion	upregulated	1.4
Slc41a2	magnesium	upregulated	1.9
