# Supplementary material for: Microbial Regulation of Glucose Metabolism and Cell-Cycle Progression in Mammalian Colonocytes
Source: PLoS One. 2012 Sep 28;7(9):e46589. doi: 10.1371/journal.pone.0046589 (PMC3460890; doi:10.1371/journal.pone.0046589)
Supplement: Table S2 — Histone Gene Expression Changes in GF Colonocytes. (PDF) [file pone.0046589.s002.pdf]

TABLE 2
Histone gene expression changes in GF colonocytes

Gene*	Histone	Status in GF	Fold change
Hist1h2aa	H2A	unchanged	N.A.
Hist1h2ab	H2A	downregulated	2.1
Hist1h2ac	H2A	unchanged	N.A.
Hist1h2ad	H2A	unchanged	N.A.
Hist1h2ae	H2A	downregulated	1.9
Hist1h2af	H2A	downregulated	1.6
Hist1h2ag	H2A	downregulated	1.7
Hist1h2ah	H2A	downregulated	2.1
Hist1h2ai	H2A	downregulated	1.7
Hist1h2ak	H2A	downregulated	1.9
Hist1h2an	H2A	downregulated	1.9
Hist1h2ao	H2A	downregulated	1.7
Hist2h2aa1	H2A	unchanged	N.A.
Hist2h2aa2	H2A	unchanged	N.A.
Hist2h2ab	H2A	unchanged	N.A.
Hist2h2ac	H2A	downregulated	1.6
Hist3h2a	H2A	unchanged	N.A.
Hist1h2ba	H2B	unchanged	N.A.
Hist1h2bb	H2B	downregulated	2.1
Hist1h2bc	H2B	unchanged	N.A.
Hist1h2be	H2B	unchanged	N.A.
Hist1h2bf	H2B	downregulated	1.5
Hist1h2bg	H2B	unchanged	N.A.
Hist1h2bh	H2B	downregulated	1.5
Hist1h2bj	H2B	downregulated	1.4
Hist1h2bk	H2B	downregulated	1.6
Hist1h2bl	H2B	downregulated	1.5
Hist1h2bm	H2B	downregulated	1.4
Hist1h2bn	H2B	downregulated	1.5
Hist1h2bp	H2B	unchanged	N.A.
Hist2h2bb	H2B	downregulated	1.4
Hist2h2be	H2B	downregulated	1.9
Hist3h2ba	H2B	unchanged	N.A.
Hist3h2bb	H2B	unchanged	N.A.
Hist1h3a	H3	unchanged	N.A.
Hist1h3b	H3	downregulated	1.9
Hist1h3c	H3	downregulated	1.8
Hist1h3d	H3	unchanged	N.A.
Hist1h3e	H3	downregulated	1.9
Hist1h3f	H3	unchanged	N.A.
Hist1h3g	H3	unchanged	N.A.
Hist1h3h	H3	unchanged	N.A.

Hist1h3i	H3	downregulated	1.9
Hist2h3b	H3	downregulated	1.7
Hist2h3ca1	H3	unchanged	N.A.
Hist2h3ca2	H3	unchanged	N.A.
Hist1h4a	H4	unchanged	N.A.
Hist1h4b	H4	unchanged	N.A.
Hist1h4c	H4	unchanged	N.A.
Hist1h4d	H4	unchanged	N.A.
Hist1h4f	H4	downregulated	1.5
Hist1h4h	H4	unchanged	N.A.
Hist1h4i	H4	unchanged	N.A.
Hist1h4j	H4	unchanged	N.A.
Hist1h4k	H4	unchanged	N.A.
Hist1h4m	H4	unchanged	N.A.
Hist2h4	H4	unchanged	N.A.
Hist4h4	H4	unchanged	N.A.
Hist1h1a	H1	unchanged	N.A.
Hist1h1b	H1	downregulated	1.9
Hist1h1c	H1	unchanged	N.A.
Hist1h1d	H1	unchanged	N.A.
Hist1h1e	H1	unchanged	N.A.
Hist1h1t	H1	unchanged	N.A.

*Genes separated by lines based on encoded histone (H2A, H2B, H3, H4, H1) and genomic cluster *(Hist1, Hist2, Hist3, Hist4).*
